# Validation of a Portuguese version of the Oral Health Impact Profile adapted to people with mild intellectual disabilities (OHIP-14-MID-PT)

**DOI:** 10.1371/journal.pone.0198840

**Published:** 2018-06-14

**Authors:** Patrícia Couto, Paulo Almeida Pereira, Manuel Nunes, Rui Amaral Mendes

**Affiliations:** 1 Faculty of Health Sciences, Beira Interior University, Covilhã, Portugal; 2 Department of Economics, Management and Social Sciences, Portuguese Catholic University, Viseu, Portugal; 3 Department of Oral and Maxillofacial Medicine and Diagnostic Sciences, School of Dental Medicine, Case Western Reserve University, Cleveland, OH, United States of America; University of Washington, UNITED STATES

## Abstract

**Background:**

The purpose of this study is to develop a Portuguese version of the Oral Health Impact Profile (OHIP-14) and validate it for people with mild intellectual disability (OHIP-14-MID-PT).

**Methods:**

The Portuguese version of the questionnaire was drawn up from the original English version, following internationally defined guidelines. Interviews were conducted with 240 individuals living in (or attending) institutions of the central region of Portugal that are affiliated with Humanitas (Portuguese Federation for Intellectual Disability) to measure oral health related quality of life (OHRQoL). The interview also included a sociodemographic and oral health questionnaire followed by an intraoral examination. Two types of reliability were analyzed: test-retest (ICC) and internal consistency (Cronbach´s α, inter-item and item-total correlations). Convergent and divergent validities were also assessed, and a confirmatory factor analysis was performed using the maximum likelihood method.

**Results:**

The OHIP-14-MID-PT presented high reliability (ICC = 0.999; Cronbach's α = 0.922). The inter-item correlation coefficient ranged from 0.277 to 0.749, and the item-total correlation coefficient varied between 0.529 and 0.718. Lower OHIP-14-MID-PT total scores were significantly associated with: a self-perception of better oral health status (r = -0.545, p<0,001) and reduced need for dental treatment (U = 2366.5, p<0,001), more natural teeth (χ^2^ = 29.74, p<0,001) and better results in the clinical oral health index (COHI) (χ ^2^ = 18.50, p<0,001); the results support the convergent and divergent validities of the questionnaire.

**Conclusions:**

OHIP-14-MID-PT has proved to be a consistent, valid and reliable instrument with good psychometric properties to determine the impact of oral health on quality of life in adults with mild intellectual disabilities in Portugal.

## Introduction

There are several definitions of intellectual disability. In this study, we opted to use the American Association on Intellectual and Developmental Disabilities (AAIDD) definition, which is the one most widely used in the literature. In the definition, intellectual disability is characterized by "significant limitations both in intellectual functioning (reasoning, learning, problem solving) and in adaptive behavior, which covers a range of everyday social and practical skills. This disability originates before the age of 18" [1].

People with intellectual disability are known to be more prone to develop oral health problems [2]. Studies indicate that individuals with intellectual disability have poorer oral hygiene [3,4], worse signs of gingival disease [2,5] and a higher prevalence of untreated dental caries [2,5–7] compared with the general population [3,8].

Oral diseases impact the functional, psychological and social dimensions of the aspects of daily routines and impair quality of life [9,10]. Furthermore, the oral health impact on overall health, nutrition and wellbeing is greater among people with special needs than in the general population, given their poorer access to oral health care [10,11].

A wide variety of quality of life evaluation tools have been developed as a result of the growing concern about the impact of oral health on an individual’s quality of life [12]. However, the studies of these tools rarely have included persons with intellectual and developmental disabilities or neurocognitive disorders [13]. One of the most widely used instruments is the Oral Health Impact Profile (OHIP) [14]. The original version, the OHIP-49, consists of 49 items representing seven domains: functional limitation, physical pain, psychological discomfort, physical disability, psychological disability, social disability and handicap.

However, several challenges led to the need to create a shortened version of the OHIP-49, including greater respondent burden and increased administrative and data management costs, resulting in low response rates when compared to shorter versions [15]. These problems led to shortened versions. One developed by Slade [16] in 1997 and a later one developed by Locker and Allen [17] in 2002. Both versions of the OHIP-14 have good psychometric properties, and have been translated and validated in different languages and populations in several countries [15,18–20]. No Portuguese version of the OHIP-14 questionnaire has been developed and validated for populations with mild intellectual disabilities.

The objective of our overall study was to 1) develop and validate a Portuguese-language version of the 14-items questionnaire of the "Oral Health Impact Profile" created by Slade and 2) to subsequently assess oral health and its impact on the quality of life of people with mild intellectual disabilities. The current paper explores and discusses the methodologies that supported the first objective, therefore providing health professionals with a suitable instrument.

## Methods

The development of a Portuguese version of the OHIP-14-MID-PT adapted to people with mild intellectual disabilities included a cross-cultural adaptation and the subsequent exploration of the psychometric properties of the scale.

### Cross-cultural adaptation of the OHIP

To develop the Portuguese version of the OHIP-14 questionnaire, the procedures for translation and adaptation of epidemiological instruments with a focus on cross-cultural and conceptual significance, previously described in the literature, were used [21,22]. The translation process of the English version was carried out by two independent Native Portuguese speaking bilingual translators. Two separate versions were obtained, that were merged by consensus. This version was translated back to English by two independent Native English speaking bilingual translators. A discussion group reviewed the original translations and the back translations looking for consensus and discrepancies. To overcome the lack of a suitable measure of reading ease for Portuguese, an expert in modern language and communication acted as a consultant. The version obtained was evaluated by the psychologists and technical directors of all participating institutions.

The pre-final version was then subjected to a pretest, to evaluate its content, formulation, sequence and average duration of application. The participants consisted of a convenience sample of 20 individuals with mild intellectual disabilities randomly selected from one of the institutions participating in the study. After each answer, the participant was asked the probe question ‘What do you mean?’ and was encouraged to expand his understanding of the item in an open-ended manner. This ensured that the final item was understood as having a meaning equivalent to that of the source item. No suggestions for changes were made.

### Sample selection and study design

All of the 13 institutions affiliated with Humanitas (Portuguese Federation for Intellectual Disability) in the central region of the country participated. Potential participants were 556 individuals with mild intellectual disabilities living in (or attending) these institutions.

Inclusion criteria were a minimum age of 18 years, having a medical report and psychological assessment attesting the condition of mild intellectual disability and authorizing participation in the study through informed consent. The sample size was calculated for a margin of error of 5%, obtaining the minimum number of 228 individuals. To avoid possible sampling biases, a significantly higher margin (288 individuals) was set to obtain the minimum sampling value. Of the 288 subjects, 240 met the inclusion criteria and were validated to reach the pre-established error estimate. Thus, a sample of 240 individuals were interviewed and clinically examined. The fifth version of the Diagnostic and Statistical Manual (DSM-5) [23], and the International Classification of Diseases (ICD-10) [24] were used to match the defining criteria of mild intellectual disability.

The data collection instruments used included sociodemographic and oral health questionnaires, the OHIP-14-MID-PT questionnaire and clinical examination guided by the Clinical Oral Health Index (COHI), the Clinical Oral Care Needs Index (COCNI) and the Clinical Oral Prevention Index (COPI) [25]. See S1 and S2 Files.

Questionnaire administration and clinical examinations were carried out by a single trained researcher. When an individual did not respond or answered a question as “don’t know”, the response was treated as a missing value and handled by item-wise deletion in the analyses.

The questionnaires were administered in the form of an interview, which allowed their application to individuals with characteristics that could affect completion, while overcoming potential illiteracy constrains. See S3 File.

In carrying out the interviews, the following methodology was used, as suggested by WHO [26]: presentation of an introduction to the study, in which the objectives and purposes of the research were explained; the questions were placed exactly as they appear in the questionnaire and in the same sequence; a neutral attitude was maintained in order not to influence the answers. Whenever the respondent showed signs of fatigue or nervousness, the interview was immediately stopped and continued later.

For the clinical exams, the examiner was calibrated. Intra-examiner reliability was verified by paired comparisons of consistency between two evaluations for 20 participants regarding the criteria and registration codes established in the COHI, COCNI, and COPI indices.

Written informed consent was obtained in the presence of care providers. The ability to provide written informed consent was determined by the clinicians of each institution or by the information available in the clinical records. Participation was confidential, voluntary and uncompensated.

This study was approved by the Ethics Committee of the Faculty of Health Sciences of the University of Beira Interior and the Ethics Committee of APPACDM—Viseu; APPACDM -Coimbra; APPACDM—Figueira da Foz; and APPACDM Vila Nova de Poiares and Arcil- Lousã. The entire study was carried out in accordance with the principles of the Helsinki Declaration (version 2013).

### OHIP-14-MID-PT

This questionnaire consists of 14 questions distributed in 7 dimensions of oral impact: functional limitation, physical pain, psychological discomfort, physical disability, psychological disability, social disability and handicap.

Each question is evaluated on a Likert scale of 5 points (never = 0, hardly ever = 1, occasionally = 2, fairly often = 3 and very often = 4). The "don’t know" option is also present. The questions relate to how often individuals have experienced each problem in the last 12 months.

### Analysis plan

#### Reliability

Two types of reliability were assessed: test-retest and internal consistency. Test-retest reliability was determined by calculating the intraclass correlation coefficient (ICC) according to the method of Shrout and Fleiss [27], using the results of a second administration of the OHIP-14-MID-PT on 20 participants, two weeks after the initial administration. The ICC was calculated for the entire scale and for each of its seven dimensions. The confidence intervals were set at 95% according to the Bland and Altman method [28]. The values defined for ICC analysis were weak <0.40, moderate 0.41–0.60, good 0.61–0.80 and excellent> 0.80 [29].

Cronbach's α was used to measure the internal consistency [30]. The impact on the α value, removing items from the OHIP-14 (α if item deleted) was evaluated, as well as the inter-item and item-total correlations.

#### Validity: Convergent validity and divergent validity

Convergent validity was assessed by identifying associations between the variables "self-perception of need for dental treatment" and "self-perception of oral health status" from the oral health questionnaire and the OHIP-14-MID-PT total score. It was then assumed that self-perception of good oral health and no need for treatment would be associated with inferior results in the OHIP-14-MID-PT total score.

For the divergent validity we compared the OHIP scores with the oral health variable—number of natural teeth—and with COHI results. It was therefore assumed that high OHIP-14-MID-PT scores would be associated with a high number of missing teeth and one or more oral problems with an important to severe health impact (COHI-2 on clinical examination).

#### Construct validity: Confirmatory factor analysis

Confirmatory factor analysis (CFA) was performed using the maximum likelihood method to verify if there was convergent validity among all dimensions as well as to verify the existence of significant relationships between them. To measure the quality of the adjustment, the reference values recommended by Maroco [31] and Arbuckle [32] were used.

## Results

### Reliability

The intraclass correlation coefficient (ICC) presented a value of ICC = 0.999 with a 95% confidence interval of 0.996–0.999, thus attaining a very high reliability [33]. The mean of the variation obtained in the two measurements for the OHIP-14-MID-PT was 0.20 ± 0.89. The ICCs of the seven dimensions of the OHIP ranged from 0.98 to 1.

There was no significant difference between the results of the two administrations (F_1,19_ = 1.000, p = 0.330).

Cronbach's α was 0.922. We also verified that the internal consistency was appropriate, given the homogeneity of values of the inter-item correlations matrix; no negative inter-item correlation was found, with results ranging from 0.277 (between ohip 3 and ohip 2) to 0.749 (between ohip 13 and ohip 12). The item-total correlation coefficients varied between 0.529 and 0.718, with the minimum correlation value being 0.529, which was well above 0.20 (the recommended minimum value to include an item on a scale) [19,34].

The removal of one item at a time resulted in lower α values, compared to the original values obtained, supporting the inclusion of all the items.

### Convergent validity

In the OHIP-14-MID-PT global scale, there were statistically significant differences (U = 2366.5, p<0.001) among those who felt they need some type of dental treatment (M = 11.89, SD = 11.50) and those who did not feel they needed treatment (M = 5.37; SD = 7.66). Therefore, OHIP values are higher for those who felt they needed dental treatment; see Table 1.

**Table 1:** Portuguese language validation of OHIP-14-MID-PT.

	Q7	N (%)	Mean	(SD)	Mann-Whitney U	P value
OHIP-14-MID-PT	No	52 (25%)	5,37	(7,66)	2366,5	< 0,001
	Yes	156 (75%)	11,89	(11,50)		
1. Functional limitation	No	56 (25,3%)	1,00	(1,87)	3920,5	0,059
	Yes	165 (74,7%)	1,35	(1,76)		
2. Physical pain	No	55 (24,4%)	1,51	(1,88)	3127,5	< 0,001
	Yes	170 (75,6%)	2,68	(2,08)		
3. Psychological discomfort	No	55 (24,9%)	,76	(1,44)	2865,5	<0,001
	Yes	166 (75,1%)	2,25	(2,35)		
4. Physical disability	No	56 (24,9%)	1,09	(1,75)	3868,5	0,027
	Yes	169 (75,1%)	1,83	(2,21)		
5. Psychological disability	No	55 (24,6%)	,45	(1,02)	2497,5	< 0,001
	Yes	169 (75,4%)	2,23	(2,26)		
6. Social disability	No	55 (24,8%)	,49	(,98)	4153,5	0,181
	Yes	167 (75,2%)	1,08	(1,95)		
7. Handicap	No	56 (25%)	,29	(,80)	3772	0,005
	Yes	168 (75%)	1,02	(1,77)		

Convergent validity: Relationship between OHIP-14-MID-PT total and subscale scores and question “7. Do you feel that you need some type of dental treatment?” (Mann Whitney Test, n = 208).

There was also a moderate negative correlation (r = -0.545, p<0.001) between the OHIP-14-MID-PT total score and the responses to question 9, “How would you describe the condition of your teeth and gums?”, which meant that individuals who presented lower OHIP results had a positive self-perception of the state of their teeth and gums and vice versa.

In addition, there were positive correlations between all dimensions scores of the OHIP-14-MID-PT scale with question 9 (p<0,001), between low correlation for dimension “1. Functional limitation” (r = -0.360) and moderate correlation for dimension “5. Psychological disability” (r = -0.551).

### Divergent validity

There were statistically significant differences in the total score (χ2 = 29.74, p<0.001) among respondents with 20 or more teeth (M = 7.34, SD = 8.90), those with 10–19 teeth (M = 14.20, SD = 12.31) and those with 1–9 teeth (M = 17.10, SD = 12.17). Those with no teeth have an average value of self-perceived quality of life identical to those who have 20 or more teeth, possibly resulting from prosthetic rehabilitation; see Table 2.

**Table 2:** Portuguese language validation of OHIP-14-MID-PT.

		N (%)	Mean	(SD)	χ^2^ (KW)	P value
OHIP-14-MID-PT	None	5 (2,3%)	8,60	(11,63)	29,74	<0,001
	1–9 teeth	31 (14%)	17,10	(12,17)		
	10–19 teeth	40 (18,1%)	14,20	(12,31)		
	20 or more teeth	145 (65,6%)	7,34	(8,90)		
1. Functional limitation	None	5 (2,1%)	1,20	(2,17)	22,87	<0,001
	1–9 teeth	32 (13,6%)	2,44	(2,05)		
	10–19 teeth	42 (17,9%)	1,57	(1,80)		
	20 or more teeth	156 (66,4%)	,88	(1,55)		
2. Physical pain	None	5 (2,1%)	1,80	(1,10)	2,62	0,454
	1–9 teeth	32 (13,4%)	2,72	(2,14)		
	10–19 teeth	42 (17,6%)	2,62	(2,16)		
	20 or more teeth	160 (66,9%)	2,22	(2,04)		
3. Psychological discomfort	None	5 (2,1%)	1,00	(2,24)	26,85	< 0,001
	1–9 teeth	31 (13,2%)	3,35	(2,42)		
	10–19 teeth	40 (17%)	2,60	(2,35)		
	20 or more teeth	159 (67,7%)	1,35	(1,95)		
4. Physical disability	None	5 (2,1%)	2,80	(3,11)	32,44	<0,001
	1–9 teeth	32 (13,4%)	2,75	(2,16)		
	10-teeth	42 (17,6%)	2,50	(2,16)		
	20 or more teeth	160 (66,9%)	1,12	(1,88)		
5. Psychological disability	None	5 (2,1%)	,60	(1,34)	15,73	0,001
	1–9 teeth	32 (13,4%)	2,72	(2,53)		
	10–19 teeth	42 (17,6%)	2,48	(2,34)		
	20 or more teeth	159 (66,8%)	1,38	(1,88)		
6. Social disability	None	5 (2,1%)	,60	(1,34)	10,56	0,014
	1–9 teeth	32 (13,6%)	1,59	(2,05)		
	10–19 teeth	42 (17,9%)	1,31	(2,10)		
	20 or more teeth	156 (66,4%)	,67	(1,53)		
7. Handicap	None	5 (2,1%)	,60	(1,34)	9,36	0,025
	1–9 teeth	32 (13,4%)	1,41	(2,11)		
	10–19 teeth	42 (17,6%)	1,24	(1,92)		
	20 or more teeth	159 (66,8%)	,55	(1,29)		

Divergent validity: OHIP-14-MID-PT scores by missing teeth (Kruskal-Wallis Test, n = 221).

There were statistically significant differences in scores among those with COHI level 2 (M = 12.61, SD = 11.63) and those with COHI level 0 or level 1 (M = 7.04, SD = 8.99) (χ2 = 18.50, p<0.001). Thus, individuals identified with code 2 in the COHI index, that is, with one or more oral problems with an important to severe health impact, present higher values ​​in the OHIP-14-MID-PT compared to those who do not present oral problems or have oral problems with a low to moderate health impact (codes 0 and 1 in the COHI index); see Table 3.

**Table 3:** Portuguese language validation of OHIP-14-MID-PT.

		N (%)	Mean	(SD)	χ^2^ (KW)	P value
OHIP-14-MID-PT	No oral health problems	5 (2,3%)	5,00	(5,10)	18,50	<0,001
	Low to moderate impact	94 (43,5%)	7,04	(8,99)		
	Important to severe impact	117 (54,2%)	12,61	(11,63)		
1. Functional limitation	No oral health problems	5 (2,2%)	1,00	(2,24)	6,19	0,045
	Low to moderate impact	100 (43,5%)	,95	(1,65)		
	Important to severe impact	125 (54,3%)	1,45	(1,80)		
2. Physical pain	No oral health problems	5 (2,1%)	2,20	(1,92)	8,78	0,012
	Low to moderate impact	100 (42,7%)	1,90	(1,89)		
	Important to severe impact	129 (55,1%)	2,72	(2,17)		
3. Psychological discomfort	No oral health problems	5 (2,2%)	,60	(1,34)	19,07	<0,001
	Low to moderate impact	100 (43,5%)	1,16	(1,79)		
	Important to severe impact	125 (54,3%)	2,42	(2,38)		
4. Physical disability	No oral health problems	5 (2,1%)	,00	(,00)	23,58	<0,001
	Low to moderate impact	101 (43,2%)	,98	(1,66)		
	Important to severe impact	128 (54,7%)	2,13	(2,25)		
5. Psychological disability	No oral health problems	5 (2,1%)	1,00	(1,41)	22,20	<0,001
	Low to moderate impact	100 (42,9%)	1,03	(1,68)		
	Important to severe impact	128 (54,9%)	2,36	(2,28)		
6. Social disability	No oral health problems	5 (2,2%)	,20	(,45)	2,88	0,237
	Low to moderate impact	98 (42,6%)	,71	(1,55)		
	Important to severe impact	127 (55,2%)	1,10	(1,91)		
7. Handicap	No oral health problems	5 (2,1%)	,00	(,00)	7,87	0,020
	Low to moderate impact	101 (43,3%)	,50	(1,18)		
	Important to severe impact	127 (54,5%)	1,06	(1,83)		

Divergent validity: OHIP-14-MID-PT scores by Clinical Oral Health Index scores (Kruskal-Wallis Test, n = 216).

### Construct validity: Confirmatory factor analysis

There was a convergent validity for all dimensions, since factor saturations were high (the lowest value was 0.620 for item OHIP 9 in the psychological disability dimension) and factor saturations were all significant (p <0.001). All dimensions also presented significant relationships (p <0.001) between themselves.

In our study, the measures indicated an acceptable adjustment of the proposed model to the data collected if we consider the chi-square (χ2/d.f = 2.796), a recommended adjustment considering CFI = 0.943, a good adjustment considering the NFI = 0.916, and a mediocre adjustment considering RMSEA = 0.087. Therefore, we can conclude that the model presented overall good adjustment indices [31].

Based on the results, we can conclude that the confirmatory factor analysis supports the use of the OHIP-14-MID-PT scale's seven dimensions.

## Discussion

This study aimed to create and evaluate the Portuguese version of the OHIP-14, in terms of validity and reliability, for use among adult population with mild intellectual disabilities. To this effect, the original English version of the OHIP-14 was translated using the forward-backward technique, pre-tested in a convenience sample and then applied to a group of the Portuguese population with mild intellectual disabilities in order to test its reliability and validity. In fact, most versions of the OHIP-14, as in our case, are based on the translation and linguistic adaptation of the original English version [15–17].

For the psychometric properties, the total ICC for the instrument under study was 0.999, which is considered excellent [27]; these results are higher than those observed in other versions [16,35–37]. To evaluate the test-retest reliability, we fixed the time interval between the two administrations of the questionnaire at two weeks, the usual time in similar situations and one that is commonly considered sufficient to avoid the influence of previous results and sufficiently short to avoid clinical changes [20,35,37].

The OHIP-14-MID-PT revealed a Cronbach α coefficient higher than the values considered standard, presenting an internal consistency superior to the original version (α = 0.88) and exceeding the minimum recommended value of 0.7. Our Cronbach α values are still similar to those found in other translated versions [18,19,35].

The adjustment of each item in the scale was investigated by removing that item and evaluating the change of Cronbach's α value in the scale. It was evident that the omission of any of the 14 items did not increase the value of Cronbach's α.

The considerable internal consistency of the instrument was also supported by the findings regarding inter-item and item-total correlations.

In fact, all the inter-item correlations were positive, and none was high enough forany item to be redundant. Regarding the item-total correlation, all the items under study revealed an adequate discriminating capacity (≥0.529), which prevents the elimination of any of the OHIP-14-MID-PT items. Similar results have been observed in others translated versions of the OHIP-14 [38].

The validity of the scale was supported by the statistically significant association found between questions aiming to subjectively evaluate individuals’ oral health status and OHIP-14-MID-PT scores. This provided evidence for the instrument’s construct validity, since it was shown that the higher the OHIP-14-MID-PT total scores, the poorer the perceived oral health status and greater the treatment needs. Other studies have found similar associations [19,38–40]. The validity was also confirmed by the scales’ ability to discriminate between groups with different oral health status, which was objectively assessed by clinical measures. It was found that the more frequent the presence of one or more problems with important to severe impact on health and tooth loss, the greater the impact on individuals’ OHRQoL. Other studies also present superior results in the OHIP questionnaire, associated with worse results in clinical examination [15,41,42] and tooth loss [43–45].

Regarding the sample size, this sample consisted of 240 individuals. Other validation studies of the OHIP-14 questionnaire present similar or even lower samples [19,46,47].

Taking into account the specificity of the sample, which included some people with illiteracy, the questionnaire was conducted in the form of an interview. This principle has been applied in several studies [12,14,17,48]. The psychometric properties of the Portuguese version of the OHIP when applied using self-administered questionnaires may have different results than those reported in this study.

Thus, the Portuguese version adapted from OHIP-14 was adequate, with good validity and reliability and with satisfactory psychometric properties, making this questionnaire a useful tool to evaluate and measure the oral quality of life of Portuguese adults with mild intellectual disability.

### Limitations

Subjecting the methodology used in the present study to critical analysis, we emphasize that the investigation is limited to people with mild intellectual disabilities. We also note that cross-cultural adaptation in a context of mild intellectual disability makes it difficult to fully understand and address the homogeneity of the concept of oral health for people in this particular group of patients. Additionally, responsiveness of the OHIP-14-MID-PT were not conducted, because this will require a longitudinal study. In this way, further research is necessary to evaluate the responsiveness of the questionnaire to clinical changes after a medical intervention. We also suggest a need for more comprehensive research to explore the epidemiology of oral health-related quality of life in people with mild intellectual disability.

Regarding the difficulties encountered, we emphasize: the lack of “gold standard” studies in Portugal that allow comparisons with other instruments to assess the quality of life in this population; and the difficulty of comparing results, given the methodological differences between the different investigations (variability in sample sizes, non-randomization of the sample, different variables to assess validity, populations with distinct characteristics, and different data collection instruments).

## Supporting information

S1 File COHI, COCNI and COPI Indexes. (PDF)

S2 File Survey questions and OHIP-14-MID-PT. (PDF)

S3 File Interview schedule. (PDF)
