# Supplementary material for: Validation of a Portuguese version of the Oral Health Impact Profile adapted to people with mild intellectual disabilities (OHIP-14-MID-PT)
Source: PLoS One. 2018 Jun 14;13(6):e0198840. doi: 10.1371/journal.pone.0198840 (PMC6002038; doi:10.1371/journal.pone.0198840)
Supplement: S2 File — (PDF) [file pone.0198840.s002.pdf]

Oral Health And Quality Of Life Questionnaire

Date __/__/____

Identification number: [ ][ ][ ] Original/Double [ ]

Institution: ________________ Location: ( ) Urban ( ) Periurban ( ) Rural

I – Personal Data:

1. Gender: ( ) Female ( ) Male

2. Age: ____

3.Years of relationship with the institution: ____

4. Type of link with the institution: (if you select the first option, go directly to question 6)

( ) Residential home
( ) Center of occupational activities – COA
( ) Professional formation
( ) Special education school
( ) Home support
( ) Resource center for inclusion – RCI

Other: _____________

5. Residence:

( ) Live alone in his own home
( ) Lives with the spouse in his or her own home
( ) Lives at home with parents/other family members
( ) Lives in the household of relatives
( ) Lives at the home of friends/host families

II – Oral Health/ Oral hygiene habits:

6. How many natural teeth do you have?

( ) None
( ) 1-9 teeth
( ) 10-19 teeth
( ) 20 or more teeth

7. Do you feel that you need some type of dental treatment?

( ) Yes
( ) No
( ) Don’t know

8. Do you use dentures?

( ) Yes
( ) No

9. How would you describe the condition of your teeth and gums or dentures?

( ) Excellent
( ) Very good
( ) Good
( ) Average
( ) Weak
( ) Very weak
( ) Don’t know

10. How often do you clean your teeth and/or dental prosthesis?

( ) Daily
( ) Occasionally
( ) Never

If you brush daily, how often do you do it per day? ______
If you brush occasionally, how often do you do it? ______

11. Select the options you use to clean your teeth and/or dentures.

( ) Electric toothbrush
( ) Manual toothbrush
( ) Toothpicks
( ) Toothpaste
( ) Dental floss
( ) Interdental brush
( ) Mouthwash
( ) Lingual scraper

Others: ______________

12. How long is it since you last saw a dentist? (if you select the "never" option, skip to question 14).

( ) Less than 6 months
( ) 6 to 12 months
( ) Between 1 and 2 years
( ) Between 2 and 5 years
( ) More than 5 years ago
( ) Never
( ) Don't know/ don't remember

13. What was the reason of your last visit to the dentist?

( ) Consultation/advise
( ) Pain or trouble with teeth, gums or mouth
( ) Treatment/ follow-up treatment
( ) Don't know/ don't remember

14. How often do you eat or drink any of the following foods, even in small quantities?

	Several times a day	Every day	Several times a week	Once a week	Several tomes a month	Seldom/never
Fresh fruit						
Biscuits, cakes, cream cakes						
Sweet pies, buns						
Jam or honey						
Chewing gum containing sugar						
Sweets/candy						
Soft drinks						
Tea with sugar						
Coffee with sugar						

15. Are you a smoker?

( ) Yes
( ) No

16. During the past 30 days, on the days you drank alcohol, how many drinks did you usually drink per day?

( ) Less than 1 drink.
( ) 1 drink
( ) 2 drinks
( ) 3 drinks
( ) 4 drinks
( ) 5 or more drinks
( ) Did not drink alcohol during the past 30 days

III – OHIP-14-MID-PT:

How often have you had the problem during the last year?

1. **Have you had trouble pronouncing any words because of problems with your teeth, mouth or dentures?**

☐ Very often ☐ Fairly often ☐ Occasionally ☐ Hardly ever ☐ Never ☐ Don't know

2. **Have you felt more difficulty in feeling the taste of food because of problems with your teeth, mouth or dentures?**

☐ Very often ☐ Fairly often ☐ Occasionally ☐ Hardly ever ☐ Never ☐ Don't know

3. **Have you had painful aching in your mouth?**

☐ Very often ☐ Fairly often ☐ Occasionally ☐ Hardly ever ☐ Never ☐ Don't know

4. **Have you found it uncomfortable to eat any foods because of problems with your teeth, mouth or dentures?**

☐ Very often ☐ Fairly often ☐ Occasionally ☐ Hardly ever ☐ Never ☐ Don't know

5. **Have you been self conscious because of your teeth, mouth or dentures?**

☐ Very often ☐ Fairly often ☐ Occasionally ☐ Hardly ever ☐ Never ☐ Don't know

6. **Have you felt nervous because of problems with your teeth, mouth or dentures?**

☐ Very often ☐ Fairly often ☐ Occasionally ☐ Hardly ever ☐ Never ☐ Don't know

7. **Have you stop eating some food because of problems with your teeth, mouth or dentures?**

☐ Very often ☐ Fairly often ☐ Occasionally ☐ Hardly ever ☐ Never ☐ Don't know

8. **Have you had to interrupt meals because of problems with your teeth, mouth or dentures?**

☐ Very often ☐ Fairly often ☐ Occasionally ☐ Hardly ever ☐ Never ☐ Don't know

9. **Have you found it difficult to relax because of problems with your teeth, mouth or dentures?**

☐ Very often ☐ Fairly often ☐ Occasionally ☐ Hardly ever ☐ Never ☐ Don't know

10. **Have you been a bit embarrassed because of problems with your teeth, mouth or dentures?**

☐ Very often ☐ Fairly often ☐ Occasionally ☐ Hardly ever ☐ Never ☐ Don't know

11. **Have you been a bit irritable with other people because of problems with your teeth, mouth or dentures?**

☐ Very often ☐ Fairly often ☐ Occasionally ☐ Hardly ever ☐ Never ☐ Don’t know

12. **Have you had difficulty doing your usual jobs because of problems with your teeth, mouth or dentures?**

☐ Very often ☐ Fairly often ☐ Occasionally ☐ Hardly ever ☐ Never ☐ Don’t know

13. **Have you felt thet your life in general has been worse because of problems with your teeth, mouth or dentures?**

☐ Very often ☐ Fairly often ☐ Occasionally ☐ Hardly ever ☐ Never ☐ Don’t know

14. **Have you been totally unable to function because od problems with your teeth, mouth or dentures**?

☐ Very often ☐ Fairly often ☐ Occasionally ☐ Hardly ever ☐ Never ☐ Don’t know
