# Supplementary material for: Validation of a Portuguese version of the Oral Health Impact Profile adapted to people with mild intellectual disabilities (OHIP-14-MID-PT)
Source: PLoS One. 2018 Jun 14;13(6):e0198840. doi: 10.1371/journal.pone.0198840 (PMC6002038; doi:10.1371/journal.pone.0198840)
Supplement: S3 File — (PDF) [file pone.0198840.s003.pdf]

Operacionalização das variáveis do questionário sociodemográfico

As variáveis que se seguem e as suas designações foram retiradas ou adaptadas do questionário “Oral Health Questionnarie For Adults” da OMS.

Dados gerais

- Data: sob a forma de dia/mês/ano, permitindo desta forma remeter a avaliação clínica ou questionário para uma data específica, caso houvesse necessidade de a mesma ser revista ou consultada.

- Número de identificação: cada indivíduo examinado teve um número de identificação associado. Este número apresenta um total de algarismos igual à totalidade da amostra em estudo. Os dígitos foram inseridos antes do início das avaliações diárias para evitar erros de contagem ou duplicação de números.

Variáveis sociodemográficas

- Instituição: instituição à qual está vinculado.

- Localização: urbana; periurbana; rural.

- Género: feminino; masculino.

- Idade: em anos completos.

- Anos de vínculo com a instituição: média de anos de vínculo com a instituição.

- Tipo de vínculo com a instituição: lar residencial; centro de atividades ocupacionais - CAO; formação profissional; escola de educação especial; apoio domiciliário; centro de recursos para a inclusão - CRI. As designações desta variável dão conta das principais tipologias de respostas sociais encontradas nas instituições em estudo.

- Residência: vive sozinho no seu próprio domicílio; vive com o cônjuge no seu próprio domicílio; vive no domicílio dos pais; vive no domicílio de outros familiares; vive no domicílio de pessoas amigas/de acolhimento. As designações desta variável resultam do contacto direto com os responsáveis de cada instituição de forma a englobar todas as possibilidades de resposta perante a realidade dos utentes de cada instituição.

Nota: as variáveis "estado civil"; "escolaridade"; "profissão" frequentemente presentes em questionários sociodemográficos foram excluídas do nosso questionário, após avaliação do mesmo por psicólogos das instituições, os quais consideraram de pouca relevância, perante a realidade e o enquadramento da população em análise.

Variáveis de saúde oral

- Número de dentes naturais: nenhum; 1-9 dentes;10-19 dentes; 20 dentes ou mais.

- Autoperceção da necessidade de tratamento dentário: o impacto subjetivo da condição oral foi determinado através de duas questões. Uma delas foi a autoperceção da necessidade de tratamento dentário, usando categorias de resposta como "sim"; "não" e "não sei".

- Utilização de prótese dentária: sim; não.

- Autoperceção do estado dos dentes e gengivas: Trata-se da segunda questão para determinar o impacto subjetivo da condição oral, estando as opções de resposta categorizadas numa escala ordinal: "excelente"; "muito bom"; "bom"; "médio"; "fraco"; "muito fraco"; "não sei".

- Frequência de higienização: diariamente; ocasionalmente; nunca.

Se realiza a escovagem diariamente, quantas vezes o faz por dia?

Se realiza a escovagem apenas ocasionalmente, com que regularidade o faz?

As designações desta variável estão relacionadas com a frequência de escovagem dentária.

- Instrumentos de higienização dentária: escova elétrica; escova manual; palitos; pasta dentífrica; fio dentário; escovilhão; elixir bucal; raspador lingual; outras. Esta variável foi retirada do questionário "Oral Health Questionnarie For Adults" da OMS, contudo, as suas opções de resposta foram modificadas e sintetizadas, englobando a questão 8 e 9 do questionário da OMS numa só questão.

- Tempo decorrido desde a última ida ao Médico Dentista: menos de 6 meses; 6 a 12 meses; entre 1 a 2 anos; entre 2 a 5 anos; há mais de 5 anos; nunca.

- Motivo da última visita ao Médico Dentista: consulta de rotina/aconselhamento médico; dor ou problemas com os dentes ou gengivas; início de tratamento ou continuidade do tratamento planeado; não sei/não me recordo.

- Tipo de alimentação: para avaliar esta variável utilizou-se uma adaptação da tabela 13 do questionário “Oral Health Questionnaire For Adults” da OMS (293).

	Várias vezes ao dia	Todos os dias	Várias vezes por semana	Uma vez por semana	Várias vezes por mês	Raramente / Nunca
Frutas frescas						
Biscoitos e bolos						
Geleias ou mel						
Pastilhas com açúcar						
Doces/Guloseimas						
Refrigerantes						
Chá com açúcar						
Café com açúcar						

- Fumador: sim; não

- Consumo de bebidas alcoólicas: por dia e tendo como referência o mês anterior: menos de 1; 1; 2; 3; 4; 5 ou mais; não consumi álcool.

QUESTIONÁRIO SOCIODEMOGRÁFICO E DE SAÚDE ORAL

Data __/__/___

Número de Identificação | | | |

Instituição: ________________ Localização: ( ) Urbana ( ) Rural

I - Dados Pessoais:

1. Género: ( ) Feminino ( ) Masculino

2. Idade: ____

3. Anos de vínculo com a instituição: ____

4. Tipo de vínculo com a instituição: (caso selecione a primeira opção, passe diretamente para a questão 6)

( ) Lar residencial
( ) Centro de atividades ocupacionais – CAO
( ) Formação profissional
( ) Escola de educação especial
( ) Apoio domiciliário
( ) Centro de recursos para a inclusão – CRI

Outro: _____________

5. Residência:

( ) Vive sozinho no seu próprio domicílio
( ) Vive com o cônjuge no seu próprio domicílio
( ) Vive no domicílio dos pais
( ) Vive no domicílio de outros familiares
( ) Vive no domicílio de pessoas amigas/de acolhimento

II – Condição Oral:

6. Quantos dentes naturais tem?

( ) Nenhum
( ) 1-9 dentes
( ) 10-19 dentes
( ) 20 dentes ou mais

7. Sente que necessita de algum tipo de tratamento dentário?

( ) Sim
( ) Não
( ) Não sei

8. Usa prótese dentária?

( ) Sim
( ) Não

9. Como descreveria o estado dos seus dentes e gengivas?

( ) Excelente
( ) Muito bom
( ) Bom
( ) Médio
( ) Fraco
( ) Muito fraco
( ) Não sei

10. Com que frequência realiza a sua higiene oral?

( ) Diariamente
( ) Ocasionalmente
( ) Nunca

Se realiza a escovagem diariamente, quantas vezes o faz por dia? ______
Se realiza a escovagem apenas ocasionalmente, com que regularidade o faz? ______

11. Selecione as opções que utiliza para limpar os seus dentes.

( ) Escova elétrica
( ) Escova manual
( ) Palitos
( ) Pasta dentífrica
( ) Fio dentário
( ) Escovilhão interdentário
( ) Elixir bucal
( ) Raspador Lingual

Outras: ______________

12. Há quanto tempo visitou pela última vez o médico dentista?

( ) Menos de 6 meses
( ) 6 a 12 meses
( ) Entre 1 a 2 anos
( ) Entre 2 a 5 anos
( ) Há mais de 5 anos
( ) Nunca

13.Qual o motivo da sua última visita ao médico dentista?

( ) Consulta de rotina/aconselhamento médico
( ) Dor ou problemas com os dentes ou gengivas

( ) Início de tratamento ou continuidade do tratamento planeado
( ) Não sei/Não me recordo

14. Com que frequência come ou bebe os seguintes alimentos, mesmo que em pequenas quantidades?

	Várias vezes ao dia	Todos os dias	Várias vezes por semana	Uma vez por semana	Várias vezes por mês	Raramente / Nunca
Frutas frescas						
Biscoitos e bolos						
Geleias ou mel						
Pastilhas com açúcar						
Doces/Guloseimas						
Refrigerantes						
Chá com açúcar						
Café com açúcar						

15. É fumador?

( ) Sim
( ) Não

16. No último mês, nos dias em que consumiu bebidas alcoólicas, quantas bebidas consumiu aproximadamente por dia?

( ) Menos de 1
( ) 1
( ) 2
( ) 3
( ) 4
( ) 5 ou mais
( ) Não consumi álcool

OHIP14-MID-PT:____

Este questionário pergunta de que forma transtornos com os seus dentes, boca ou próteses podem causar-lhe problemas no seu dia-a-dia.

Responda às questões em baixo (colocando uma cruz atrás da opção que acha mais adequada) tendo em conta a frequência em que sentiu cada um dos aspetos nos **últimos 12 meses**.

1. **Teve alguma dificuldade em pronunciar algumas palavras devido a problemas com os seus dentes, boca ou prótese dentária?**

☐Quase sempre ☐Algumas vezes ☐Poucas vezes ☐ Raramente ☐ Nunca ☐ Não sei

2. **Notou mais dificuldade em sentir o sabor dos alimentos devido a problemas com os seus dentes, boca ou prótese dentária?**

☐Quase sempre ☐Algumas vezes ☐Poucas vezes ☐ Raramente ☐ Nunca ☐ Não sei

3. **Teve alguma dor na sua boca?**

☐Quase sempre ☐Algumas vezes ☐Poucas vezes ☐ Raramente ☐ Nunca ☐ Não sei

4. **Sentiu algum desconforto ao comer algum alimento devido a problemas com os seus dentes, boca ou prótese dentária?**

☐Quase sempre ☐Algumas vezes ☐Poucas vezes ☐ Raramente ☐ Nunca ☐ Não sei

5. **Tem-se sentido pouco à vontade por causa dos problemas com os seus dentes, boca ou prótese dentária?**

☐Quase sempre ☐Algumas vezes ☐Poucas vezes ☐ Raramente ☐ Nunca ☐ Não sei

6. **Sentiu-se nervoso(a) devido a problemas com os seus dentes, boca ou prótese dentária?**

☐Quase sempre ☐Algumas vezes ☐Poucas vezes ☐ Raramente ☐ Nunca ☐ Não sei

7. **Deixou de comer algum alimento devido a problemas com os seus dentes, boca ou prótese dentária?**

☐Quase sempre ☐Algumas vezes ☐Poucas vezes ☐ Raramente ☐ Nunca ☐ Não sei

8. **Teve de interromper refeições devido a problemas com os seus dentes, boca ou prótese dentária?**

☐Quase sempre ☐Algumas vezes ☐Poucas vezes ☐ Raramente ☐ Nunca ☐ Não sei

9. **Sentiu dificuldade em relaxar/descansar devido a problemas com os seus dentes, boca ou prótese dentária?**

☐Quase sempre ☐Algumas vezes ☐Poucas vezes ☐ Raramente ☐ Nunca ☐ Não sei

10. **Tem-se sentido um pouco envergonhado(a) devido a problemas com os seus dentes, boca ou prótese dentária?**

☐Quase sempre ☐Algumas vezes ☐Poucas vezes ☐ Raramente ☐ Nunca ☐ Não sei

11. **Tem sido menos tolerante ou paciente com o(a) seu(sua) companheiro(a) ou família devido a problemas com os seus dentes, boca ou prótese dentária?**

☐Quase sempre ☐Algumas vezes ☐Poucas vezes ☐ Raramente ☐ Nunca ☐ Não sei

12. **Teve dificuldade em realizar as suas atividades habituais por causa de problemas com os seus dentes, boca ou prótese dentária?**

☐Quase sempre ☐Algumas vezes ☐Poucas vezes ☐ Raramente ☐ Nunca ☐ Não sei

13. **Sentiu que a sua vida em geral tem corrido pior devido a problemas com os seus dentes, boca ou prótese dentária?**

☐Quase sempre ☐Algumas vezes ☐Poucas vezes ☐ Raramente ☐ Nunca ☐ Não sei

14. **Tem-se sentido completamente incapacitado devido a problemas com os seus dentes, boca ou prótese dentária**?

☐Quase sempre ☐Algumas vezes ☐Poucas vezes ☐ Raramente ☐ Nunca ☐ Não sei
